# Experiments on Antimony

**Published:** 1846-11

**Authors:** 


*Experiments on Antimony.*—M. E. Millon has forwarded to the Institute a series of experiments which prove, that not only the drug remains for a long time in the system after it has ceased to be exhibited, but also that it exercises a special action on the chylopoietic organs. In the dog submitted to observation, the liver increased to three times its natural size; and one bitch took tartar emetic during five days only, and a fortnight afterwards littered several living pups in the livers of which antimony was detected in large proportions.—*Lond. Med. Times.*

